# Cognitive flexibility and its electrophysiological correlates in Gilles de la Tourette syndrome

**DOI:** 10.1016/j.dcn.2017.08.008

**Published:** 2017-08-18

**Authors:** Florian Lange, Caroline Seer, Kirsten Müller-Vahl, Bruno Kopp

**Affiliations:** a Department of Neurology, Hannover Medical School, Hannover, Germany; b Behavioral Engineering Research Group, KU Leuven, Leuven, Belgium; c Movement Control & Neuroplasticity Research Group, KU Leuven, Leuven, Belgium; d Clinic of Psychiatry, Social Psychiatry and Psychotherapy, Hannover Medical School, Hannover, Germany

**Keywords:** Gilles de la Tourette syndrome, Executive functioning, Cognitive flexibility, Wisconsin card sorting test, Event-related potentials

## Abstract

Motor symptoms in Gilles de la Tourette syndrome (GTS) have been related to changes in frontostriatal brain networks. These changes may also give rise to alterations in cognitive flexibility. However, conclusive evidence for altered cognitive flexibility in patients with GTS is still lacking. Here, we meta-analyzed data from 20 neuropsychological studies that investigated cognitive flexibility in GTS using the Wisconsin Card Sorting Test (WCST). Results revealed medium-sized GTS-related performance deficits, which were significantly modulated by age: Whilst being substantial in children and adolescents with GTS, WCST deficits seem to dissolve in adult patients with GTS. This age-related normalization of WCST performance might result from the compensatory recruitment of cognitive control in adult patients with GTS. We addressed this possibility by examining neural correlates of proactive and reactive cognitive control in an event-related potential (ERP) study. We analyzed cue- and target-locked ERPs from 23 adult patients with GTS and 26 matched controls who completed a computerized version of the WCST. Compared to controls, patients with GTS showed a marked increase in parietal cue-locked P3 activity, indicating enhanced proactive cognitive control. We conclude that the additional recruitment of proactive cognitive control might ensure flexible cognitive functioning in adult patients with GTS.

## Highlights

• Gilles de la Tourette syndrome (GTS) may involve cognitive inflexibility.
• A meta-analysis reveals GTS-related deficits on the Wisconsin Card Sorting Test.
• Card-sorting deficits are larger in children than in adults with GTS.
• Adults with GTS show electrophysiological signs of enhanced cognitive control.
• This change may underlie the normalization of cognitive flexibility in adult GTS.

## 1 Introduction

Approximately one percent of school-aged children show a combination of motor and vocal tics that is commonly referred to as Gilles de la Tourette syndrome (GTS; Robertson, 2008). Many of these children continue to have tics as adults, although often with decreased severity (Pappertet al., 2003). Tics in GTS have been attributed to alterations in the basal ganglia and the associated frontostriatal circuits (Mink, 2001). In accordance with this notion, imaging studies have revealed reduced gray matter volume in the basal ganglia of both children and adults with GTS, as well as structural changes in areas of the frontal cortex (Plessen et al., 2009). Frontal cortical changes are not restricted to motor and premotor regions but also extend to the prefrontal cortex (Müller-Vahl et al., 2009). The prefrontal cortex is connected with the basal ganglia via frontostriatal circuits that are thought to be critical for efficient executive functioning (Frank et al., 2001, Hazy et al., 2007, Monchi et al., 2006, Owen, 2004, Robbins and Cools, 2014). It thus appears plausible to assume that GTS is not only associated with motor symptoms (i.e., with tics) but also with deficits in the domain of executive functioning (Eddy et al., 2012).

One central aspect of executive functioning is cognitive flexibility (Miyake et al., 2000). Cognitive flexibility has been variably defined: Some authors conceptualize cognitive flexibility as a well-delimited mental ability while others think of it more as a property of the cognitive system or a metacognitive state (Ionescu, 2012). Here, we adopt an operational definition according to which cognitive flexibility refers to the cognitive processes that allow for the efficient adaptation of goal-directed behavior to changing environmental demands (Garcia-Garcia et al., 2010).

A number of neuropsychological tests have been developed for the assessment of cognitive flexibility, the most popular of which is the Wisconsin Card Sorting Test (WCST; Berg, 1948, Grant and Berg, 1948, Heaton et al., 1993). On the WCST, participants are required to sort cards and to use the experimenter’s feedback to shift between different sorting rules. The analysis of WCST performance typically focusses on the number of completed categories and an index of perseverative tendencies (i.e., number/percentage of perseverative errors/responses). Performance on the WCST appears to be sensitive to prefrontal lobe damage (Demakis, 2003, Milner, 1963) as well as to lesions (Eslinger and Grattan, 1993) and deep brain stimulation (Jahanshahi et al., 2000, Jahanshahi et al., 2014, Pillon et al., 2006) of the basal ganglia. In addition, cognitive inflexibility on the WCST has been observed in a number of neurological and psychiatric conditions that are associated with frontostriatal dysfunction, including Parkinson’s disease (Kudlicka et al., 2011, Lange et al., 2016c), dystonia (Lange et al., 2016b, Lange et al., 2016d), and obsessive-compulsive disorder (OCD; Shin et al., 2014).

With respect to GTS, Eddy et al. (2009) conducted a review of the studies that analyzed performance on the WCST. They reported that the majority of studies did not find pronounced WCST performance deficits in patients with GTS. However, this lack of evidence for GTS-related impairment on the WCST may not be taken as evidence for intact WCST performance in these patients (Altman and Bland, 1995). Low statistical power due to small sample sizes in individual studies poses a problem to many research areas including neuropsychology (Bezeau and Graves, 2001, Demakis, 2006). This implies that, even in the presence of relevant WCST performance deficits in the population of patients with GTS, these deficits may often go undetected in individual studies. The meta-analytical aggregation of evidence across multiple neuropsychological studies allows overcoming this limitation.

In the following, we present a meta-analysis that aimed at obtaining a reliable effect-size estimate for potential WCST deficits in patients with GTS. In addition, this meta-analytical approach enabled us to investigate whether the extent of GTS-related alterations in WCST performance is moderated by patient characteristics such as age and gender. Following the description of this meta-analysis, we present an event-related potential (ERP) study that we conducted to examine the electrophysiological correlates of WCST performance in patients with GTS. In this study, we analyzed the ERP waveforms evoked by stimuli in a computerized version of the WCST. This approach allowed us to further elucidate the neural underpinnings of WCST performance in patients with GTS. Specifically, we were able to investigate whether patients with GTS differ from healthy control participants with regard to the neural correlates of the cognitive control processes that they recruit while performing the WCST. Hence, our two studies complement each other in providing (a) a powerful test of potential GTS-related WCST performance deficits (meta-analysis) and (b) an in-depth analysis of the mechanisms that underlie WCST performance in patients with GTS (ERP study).

## 2 Meta-analytic evidence for impaired WCST performance in GTS

### 2.1 Methods

**Fig. 1:**
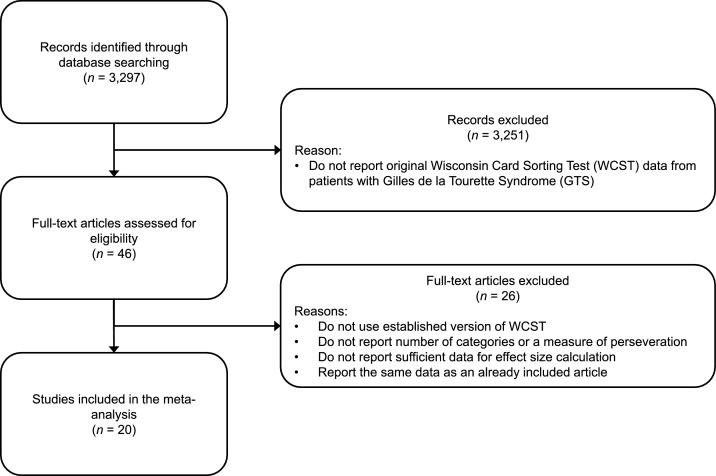
Flow chart depicting the selection of articles for our meta-analysis.

A systematic literature review was conducted in April 2015 and updated in July 2017 using the databases PubMed, ScienceDirect, PsychInfo, and Scopus as well as Google Scholar. In a first step, we screened the results of a Google-Scholar search involving the combination of the keywords “Tourette” and “Wisconsin Card Sorting Test” (2345 hits). We then looked for studies that did not explicitly mention the term “Wisconsin Card Sorting Test”, but that involved the keyword “Tourette” as well as either “Card Sort”; “Card Sorting”; “WCST”; or “MCST” (952 hits). When screening these 3297 records for eligibility; we excluded a record as soon as we were able to determine that it does not fulfill all the inclusion criteria of our meta-analysis (see Fig. 1). The following inclusion criteria were applied:

1) The study had to administer a standard version of the WCST to a sample of patients with GTS. This implies that studies reporting WCST data from single patients with GTS were not considered.

2) The study had to involve WCST performance data from a sample of healthy control participants or it had to report standard scores that describe the performance of patients with GTS in comparison to normative data.

3) The study had to report data for at least one of the best-established WCST measures (i.e., number of completed categories, percent/number of perseverative errors/responses) at a level of detail that allows for the calculation of effect sizes (i.e., test statistic, means and standard deviations, or descriptive data (median, range, interquartile range) that allow estimating means and standard deviations according to the procedure described by Wan et al., 2014).

When the title of a record did not allow determining that one of these criteria was not fulfilled, we screened the abstract. When the abstract did not allow excluding the record, we accessed the full text. After screening the potentially eligible full texts, we retained 20 records that fulfilled the criteria listed above. We repeated the same procedure using the databases PubMed, ScienceDirect, PsychInfo, and Scopus, but theses searches did not render any additional studies to be included.

Hence, we performed our meta-analysis on 20 studies reporting WCST performance data from patients with GTS. Fourteen studies involved a direct comparison between patients with GTS and healthy controls, whereas the other six studies reported standard scores based on normative data. For the studies comparing patients with GTS and healthy controls, the *t*-statistic for the between-group comparison was calculated using the two-sample *t*-test provided by GraphPad QuickCalcs (http://www.graphpad.com/quickcalcs/ttest1/). For the studies reporting standard scores, the *t*-statistic was obtained by computing a one-sample *t*-test (http://www.graphpad.com/quickcalcs/oneSampleT1/) comparing the mean standard score in the patient sample to the mean in the normative sample.

Effect sizes (Cohen’s *d*) and their confidence intervals were calculated from *t*-statistics using the syntaxes provided by Wuensch (2012). Effect sizes were transformed such that more positive values indicate more pronounced deficits in patients with GTS. When a study involved more than one group of patients with GTS, data were pooled across groups. For studies without a control group, control group size was imputed with the average control group size of all other studies.

When provided, we extracted the data for two measures of WCST performance from each study: the number of completed categories and a measure of perseveration (i.e., percent/number of perseverative errors/responses). When more than one measure of perseveration was reported (e.g., the number of perseverative errors and the number of perseverative responses, Sung and Park, 2000), only the number of perseverative errors was extracted as measure of perseveration for the particular study. Mean effect sizes and confidence intervals for both WCST measures were calculated using the random-effects model syntax provided by Field and Gillett (2010). Heterogeneity of effect sizes was examined using Cochran’s *Q* and the *I*^2^ index (Higgins et al., 2003). In addition, we tested whether effect sizes were moderated by sample characteristics (i.e., age and gender). Potential moderating effects were examined using weighted multiple regression analysis (Field and Gillett, 2010) with age (children/adolescents vs. adults) as a categorical predictor and gender (i.e., the proportion of female participants in the patient sample) as a continuous predictor. We decided to treat age as categorical predictor because the distribution of mean age across the studies was clearly bimodal. Eleven studies reported a mean age ranging from 9 to 13 years, whereas eight studies reported a mean age ranging from 29 to 41 years. One study (Matsuda et al., 2012) that included adolescent and adult participants (mean age = 18 years) was excluded from the analysis of the moderating effect of age. The Kendall’s tau rank correlation between effect sizes and their standard errors was calculated to evaluate potential publication bias (Rothstein et al., 2005).

### 2.2 Results

**Fig. 2:**
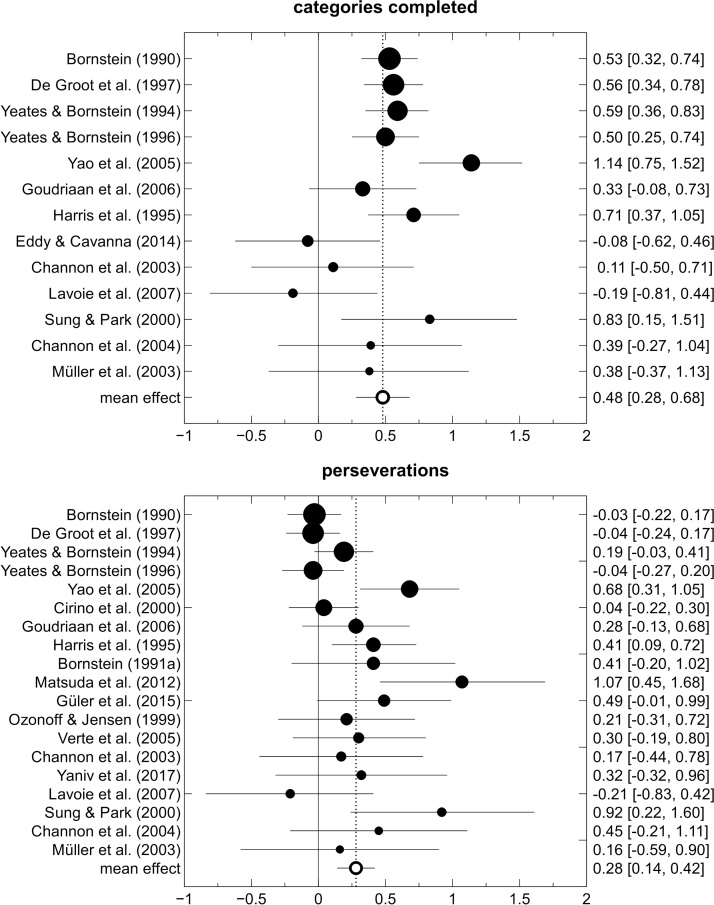
Forest plot of the effect sizes from the studies reporting Wisconsin Card Sorting Test performance data of patients with Gilles de la Tourette syndrome. Horizontal lines represent 95% confidence intervals. The area of the circles is proportional to the studies’ patient sample size.

Table 1 provides an overview of the 20 studies that were included in our meta-analysis on WCST performance in patients with GTS. Effect sizes and the corresponding confidence intervals for the individual studies are displayed in Fig. 2. Table 2 presents the results of the meta-analysis across these studies. Overall, GTS was associated with significant performance deficits on the WCST. Patients with GTS completed fewer categories (*d* = 0.48) and showed a more pronounced tendency to perseverate (*d* = 0.28) in comparison to healthy controls or to normative values. Both effect sizes remained significant when we excluded those studies that did not involve a control group. Effect-size heterogeneity for the number of completed categories was significant and moderate in size (*I*^2^ = 50%). Effect-size heterogeneity for the number of perseverations was not significant and moderate-to-small in size (*I*^2^ = 32%). Rank-correlation analysis did not reveal any signs of publication bias (both *t_b_*  < 0.21, both *p* > 0.22).

**Table 1:** Overview of the studies included in the meta-analysis of Wisconsin Card Sorting Test (WCST) performance in patients with Gilles de la Tourette syndrome.

Study	*N*_controls_	*N*_patients_	%_female_	age	extracted WCST measures
Bornstein (1990)	–	100	14	12	categories, perseverative errors
Bornstein (1991a)	15	36	NA	33	perseverative errors
Channon et al. (2003)	21	21	19	33	categories, perseverative errors
Channon et al. (2004)	23	15	22	34	categories, perseverative errors
Cirino et al. (2000)	–	57	13	12	categories, perseverative errors
De Groot et al. (1996)	–	92	15	12	categories, perseverative errors
Eddy and Cavanna (2014)	25	27	24	30	categories
Goudriaan et al. (2006)	50	46	30	37	categories, percent perseverative responses
Güler et al. (2015)	32	31	44	13	percent perseverative errors
Harris et al. (1995)	–	42	10	11	categories, perseverative errors
Lavoie et al. (2007)	22	18	55	41	categories, perseveration*
Matsuda et al. (2012)	18	33	33	18	percent perseverative errors
Müller et al. (2003)	14	14	7	29	categories, perseverative errors
Ozonoff and Jensen (1999)	29	30	NA	13	perseverative responses
Sung and Park (2000)	18	18	0	9	categories, perseverative errors
Verté et al. (2005)	47	24	15	10	percent perseverative responses
Yaniv et al. (2017)	19	19	42	29	percent perseverative errors
Yaniv et al. (2017)	60	60	17	10	categories, perseverative errors
Yeates and Bornstein (1994)	–	82	17	12	categories, perseverative errors
Yeates and Bornstein (1996)	–	70	14	12	categories, perseverative errors

Note. The study by Matsuda et al. (2012) was not included in the analysis of the moderating effect of age because it included adolescent and adult participants.%_female_ = proportion of female participants in the patient group, age = mean age of participants in the patient group in years, NA = data not available, *measure of perseveration unspecified.

**Table 2:** Results of the meta-analysis of Wisconsin Card Sorting Test performance in Gilles de la Tourette syndrome.

	WCST measure	
	Categories completed	Perseverations
Children/adolescents		
Number of studies	7	11
Number of patients	428	606
Effect size [95% CI]	0.70 [0.51, 0.89]	0.26 [0.08, 0.44]
*Q*	7.21	16.23
*I*^2^ (%)	16.78	32.22

Adults		
Number of studies	6	7
Number of patients	141	169
Effect size [95% CI]	0.17 [−0.06, 0.40]	0.23 [0.01, 0.45]
*Q*	3.43	2.85
*I*^2^ (%)	0	0

All studies		
Number of studies	13	19
Number of patients	569	808
Effect size [95% CI]	0.48 [0.28, 0.68]	0.28 [0.14, 0.42]
*Q*	24.11*	25.86
*I*^2^ (%)	50.23	32.34

Note. The study by Matsuda et al. (2012) was not included in the analysis of the moderating effect of age. As a consequence, for the analysis of perseverations, the effect size across all studies is smaller than the effect sizes in both age groups. *p < 0.05.

As can be seen from inspection of Table 2, the GTS-related decrease in the number of completed categories was significantly moderated by the age of the study sample, χ^2^(1) = 13.46, *p* = 0.001. The effect size in the adult samples is small (*d* = 0.17) with a 95% confidence interval that includes zero. The effect size in children and adolescents is large (*d* = 0.70) with a 95% confidence interval that neither includes zero, nor overlaps with the 95% confidence interval around the effect size in the adult population. Note further that effect-size heterogeneity (as measured by the *I^2^* statistic) was considerably smaller in these two subgroups than in the overall sample of studies. The effect size also appeared to be moderated by the proportion of female participants in the patient sample, β = −1.90, *p* = 0.043. However, when both predictors were entered simultaneously, only age, χ^2^(1) = 6.02, *p* = 0.014, but not gender, β = −0.53, *p* = 0.573, emerged as a significant moderator of the GTS-related decrease in the number of completed WCST categories. Neither age, χ^2^(1) = 0.03, *p* = 0.866, nor gender β = −0.50, *p* = 0.524, significantly moderated the GTS-related increase in perseveration on the WCST.

One question that cannot be adequately addressed by our meta-analysis relates to the association between WCST performance deficits and tic severity in patients with GTS. We did not find any reports of a significant correlation between these two variables in the published literature. Seven of the studies reviewed above described the absence of such a correlation and only three of them reported the magnitude of the correlation coefficients. To further complicate the interpretation of these results, the signs of the published correlation coefficient are not unambiguous, thus rendering the sensible aggregation of effect sizes across studies infeasible.

We initially planned to also analyze whether WCST performance deficits in patients with GTS are moderated by the presence of comorbid disorders (such as attention deficit (hyperactivity) disorder, AD(H)D, or OCD). However, information with regard to these comorbidities was not reported consistently across the studies involved in our meta-analysis and only five studies explicitly excluded patients with comorbidities or provided data from a subgroup of patients without comorbidities. Notably, the above-described GTS-related decrease in the number of completed WCST categories also emerged in this subset of studies including patients without comorbid disorders (categories; *k* = 5, *d* = 0.35, 95% CI = [0.03–0.68]; perseverations: *k* = 4, *d* = 0.29, 95% CI = [-0.21–0.78]).

In addition, our literature review revealed six studies that directly compared patients with GTS with and without ADD/ADHD (Cirino et al., 2000, De Groot et al., 1996, Harris et al., 1995, Ozonoff and Jensen, 1999, Schuerholz et al., 1996, Yeates and Bornstein, 1994). The studies by Ozonoff and Jensen (1999) and Schuerholz et al. (1996) did not report the information necessary to calculate effect sizes but only that the groups did not differ significantly with regard to their WCST performance. Average effect sizes across the remaining four studies were close to zero (categories: *k* = 4, *d* = 0.00, 95% CI = [−0.27–0.26]; perseverations: *k* = 4, *d* = −0.02, 95% CI = [−0.28–0.25]), indicating that there is no evidence for a contribution of ADHD symptoms to the WCST performance deficits that can be found in patients with GTS.

We did not find a similar number of studies comparing patients with GTS with and without OCD. Patients with GTS and comorbid OCD showed larger WCST performance deficits than patients without this comorbidity in the study by De Groot et al. (1996). Five additional studies examined the correlation between WCST performance and OCD symptoms in patients with GTS (Bornstein, 1991b, Eddy and Cavanna, 2014, Güler et al., 2015, Lavoie et al., 2007, Matsuda et al., 2012). Three of these studies did not find a significant relationship between the two variables, whereas Bornstein (1991b) described an association between OCD symptoms and WCST performance in patients with GTS. Moreover, the study by Matsuda and colleagues reported that patients with GTS who scored high on a particular OCD dimension (aggression) showed more pronounced WCST deficits than patients who scored low on this dimension. With regard to other comorbidities, no significant correlations have been found between WCST performance and affective symptoms (Eddy and Cavanna, 2014, Lavoie et al., 2007) and WCST performance did not differ significantly as a function of the presence of impulse control disorders (Eddy and Cavanna, 2014), high-functioning autism (Verté et al., 2005), Asperger syndrome (Berthier et al., 1993), or learning disorders (Yeates and Bornstein, 1996). Hence, at present, there is little empirical support for the notion that GTS-related WCST deficits can be attributed to the presence of comorbid disorders. While some data suggest that comorbid OCD symptoms might aggravate WCST deficits in patients with GTS, impaired WCST performance can also be found in patients with “pure GTS”.

## 3 Electrophysiological correlates of WCST performance in patients with GTS

Our meta-analysis revealed medium-sized WCST performance deficits in patients with GTS, which seem to be most pronounced with regard to the number of completed categories. However, these deficits appeared to be largely restricted to the underage population. These findings raise the question of why the pronounced WCST performance deficits in children and adolescents with GTS are markedly reduced in studies including older patients. We approached this question by analyzing the electrophysiological correlates of WCST performance in a sample of adult patients with GTS. Specifically, we applied the event-related potential (ERP) technique to explore whether adult individuals with and without GTS differ with regard to the neural processes they recruit when completing the WCST.

ERPs can be obtained by averaging EEG activity that is time-locked to the stimuli or responses occurring during the course of a laboratory task. The voltage deflections comprising the ERP are commonly thought to be linked to the neural mechanisms underlying cognitive processes (Duncan et al., 2009). In the last fifteen years, the ERP technique has been applied to the study of GTS-related deficits in various executive functions including performance monitoring (Eichele et al., 2016, Hanna et al., 2012, Johannes et al., 2002), inhibitory control (Johannes et al., 2001a, Lavoie et al., 2011, Shephard et al., 2016a), conflict resolution (Johannes et al., 2003, Morand-Beaulieu et al., 2015, Thibault et al., 2009), feedback processing (Shephard et al., 2016b), and dual tasking (Johannes et al., 2001b). Across these studies, ERPs were particularly useful in revealing insights into the cognitive processing of task events that do not require overt motor responses. Such processes (e.g., the processing of errors or of signals that require the participant to withhold responding) are not accessible via the analysis of performance measures such as response times (RTs). They are, however, associated with characteristic signatures in the ERP.

In contrast to the broad interest in the executive functions listed above, no study has yet investigated the electrophysiological correlates of cognitive flexibility as it is required on the WCST in patients with GTS. One possibility to measure these correlates is to combine the ERP technique with a computerized version of the WCST (Barceló, 1999, Barceló, 2003, Barceló et al., 2000, Barceló et al., 2002, Barceló et al., 1997, Kopp and Lange, 2013, Mattes et al., 1991, Vilà-Balló et al., 2015). When used as a behavioral task, the computerized WCST (cWCST) allows assessing participants’ overt responses to target events to examine the latency and accuracy of the card-sorting process (Lange et al., 2016a). When combined with the ERP technique, the cWCST also allows studying the neural responses to feedback cues that instruct participants whether to repeat or shift the previously applied sorting rule (Barceló, 2003, Cunillera et al., 2012). By this means, the ERP technique allows to dissociate proactive (i.e., cue-related) and reactive (i.e., target-related) cognitive control processes that jointly contribute to performance on the cWCST (Adrover-Roig and Barceló, 2010).

In shifting paradigms (such as the cWCST), proactive and reactive control processes have been proposed to manifest themselves in modulations of ERP deflections in the latency range of the P3 (250–500 ms after stimulus onset; (Kopp et al., 2014). Cue-locked P3 amplitudes are typically larger in response to shift cues (i.e., feedback stimuli signaling that the rule has to be shifted) than in response to repeat cues (i.e., feedback stimuli signaling that the rule has to be maintained; (Barceló et al., 2002, Barceló et al., 2006, Gajewski and Falkenstein, 2011, Kopp and Lange, 2013, Kopp et al., 2006). Cue-locked P3 activity in shifting paradigms likely reflects proactive cognitive processes such as the preparatory activation of task rules (Barceló et al., 2002). In contrast, the target stimuli following a shift cue typically elicit smaller P3 amplitudes than the target stimuli following a repeat cue (Barceló et al., 2000, Barceló et al., 2002, Gajewski and Falkenstein, 2011) and target-locked P3 activity in shifting paradigms appears to be linked to reactive cognitive control processes (Hsieh and Cheng, 2006, Jamadar et al., 2010, Tarantino et al., 2016).

Through the combination of a computerized version of the WCST and the ERP technique, we examined whether adult patients with GTS show alterations in the neural correlates of proactive and/or reactive processes contributing to cognitive flexibility. By analyzing these distinct modes of cognitive control (Braver et al., 2007) we aimed to generate some first insights into the mechanisms underlying the age-dependent normalization of WCST performance in patients with GTS.

### 3.1 Methods

#### 3.1.1 Participants

Twenty-three patients with GTS (10 female, 13 male, *M*_age_ = 32.78 yrs, *SD*_age_ = 11.11 yrs) were tested between June 2014 and July 2015. The diagnosis of GTS was confirmed by an experienced psychiatrist (KMV) according to DSM-5. Eight patients were on psychiatric medication on the day of testing (aripiprazole: *n* = 3, citalopram: *n* = 1, sertraline: *n* = 1, agomelatine: *n* = 1, methylphenidate: *n* = 1, risperidone: *n* = 1, tetrahydrocannabinol: *n* = 1).

Twenty-six adults (11 female, 15 male, *M*_age_ = 32.88 yrs, *SD*_age_ = 11.23 yrs) without psychiatric and neurological diseases served as control participants. One additional control participant was tested but excluded from all analyses due to extremely slow RTs (i.e., deviating more than three SDs from the mean of the control group) on the cWCST. The group of control participants did not differ significantly from the GTS group in terms of age, gender, or the number of education years, all *p >* 0.79, see Table 3. Patients and controls had to be at least 18 years old to be included in the study. All participants were offered a compensation of 25 € for their participation. The study was approved by the ethics committee of Hannover Medical School (vote number: 6589). All participants gave informed consent in accordance with the Declaration of Helsinki.

**Table 3:** *Demographic* and psychological characteristics of the included patients with Gilles de la Tourette syndrome (GTS) and control participants.

	GTS	controls		
	Mean (SD)	Mean (SD)	*t*	*p*
Age (years)	32.78 (11.11)	32.88 (8.43)	−0.36	0.971
Education (years)	14.54 (3.45)	14.31 (2.68)	0.26	0.799
WST	28.39 (7.65)	27.92 (5.77)	0.24	0.810
MoCA	27.96 (1.87)	28.62 (1.30)	−1.45	0.155

M-WCST				
completed categories	5.87 (0.46)	6.04 (0.34)	−1.47	0.148
perseverative errors	0.26 (0.54)	0.38 (0.75)	−0.65	0.517
BIS-Brief	15.26 (3.66)	14.71 (3.00)	0.57	0.573
BDI-II	11.96 (9.83)	6.08 (5.23)	2.54	0.016

BSI-18				
Global severity	11.87 (11.89)	4.42 (4.83)	2.79	0.009
Anxiety	4.74 (5.15)	1.92 (2.38)	2.39	0.023
Depression	3.83 (4.52)	1.29 (1.68)	2.53	0.017
Somatization	3.30 (4.35)	1.21 (1.98)	2.11	0.043

CAARS (t-values)				
inattention	46.17 (7.88)	42.75 (6.74)	1.60	0.116
hyperactivity	48.26 (9.93)	43.79 (7.36)	1.76	0.085
impulsivity	49.61 (11.41)	43.54 (8.20)	2.10	0.041
self-concept	51.09 (10.30)	43.21 (6.14)	3.20	0.003
inattentive symptoms	48.61 (12.42)	42.00 (9.16)	2.08	0.043
hyperactive-impulsive symptoms	49.74 (13.03)	43.63 (8.25)	1.93	0.060
ADHD symptoms	49.43 (12.54)	42.54 (9.42)	2.14	0.038
ADHD index	50.91 (10.04)	43.46 (8.74)	2.72	0.009
WURS-k	23.26 (13.92)	15.88 (16.82)	1.64	0.109

DSM-IV list				
attention	4.09 (2.70)	2.00 (2.73)	2.61	0.012
hyperactivity	4.09 (3.15)	1.92 (2.55)	2.58	0.013

Note. One control participant completed only the MoCA while another control participant completed only the MoCA and the WST. As a result, control sample size was *n* = 24 instead of *n* = 26 for the remaining measures. WST = Wortschatztest (German vocabulary test of premorbid intelligence); MoCA = Montreal Cognitive Assessment; M-WCST = Modified Wisconsin Card Sorting Test; BIS-Brief = Barratt Impulsiveness Scale − Brief; BDI-II = Beck’s Depression Inventory II; BSI–18 = Brief Symptom Inventory (18-item version); CAARS=Conners Adult ADHD Rating Scale; WURS-k = Wender Utah Rating Scale − short form; DSM-IV list = DSM-IV symptom list for attention deficit hyperactivity disorder.

#### 3.1.2 Background assessment and comorbidities

A number of clinical and (neuro-) psychological assessments were administered to further characterize our sample of patients with GTS and healthy controls. Some measures were only administered to patients with GTS. Disease severity was quantified using the Yale Global Tic Severity Scale (YGTSS; Leckman et al., 1989; *M* = 22.52, *SD* = 11.10) and the Adult Tic Questionnaire (ATQ; Abramovitch et al., 2015; *M* = 50.60, *SD* = 40.57). Patients also completed the Premonitory Urge for Tics Scale (PUTS; Woods et al., 2005; *M* = 21.22, *SD* = 7.93). The Yale-Brown Obsessive Compulsive Scale (Y-BOCS; Goodman et al., 1989; *M* = 8.22, *SD* = 9.72) was administered to confirm the clinical diagnosis of OCD (*M* = 8.22, *SD* = 9.72). Three patients had been diagnosed with OCD based on the clinical assessment of an experienced psychiatrist (KMV). These diagnoses were confirmed by Y-BOCS scores ≥ 16 in all these three patients.

Other measures were administered to both patients and control participants (see Table 3). Some of these measures were included to assess whether patients fulfilled the diagnostic criteria for ADHD, a common comorbidity of GTS. These measures include the DSM-IV symptom list for ADHD (Rösler et al., 2004), a short form of the Wender Utah Rating Scale (WURS-k; Retz-Junginger et al., 2002, Ward et al., 1993), and the Conners Adult ADHD Rating Scale (CAARS; Christiansen et al., 2011). Data from these three measures were combined for the assessment of current ADHD. T-scores ≥ 65 on at least four of the eight subscales of the CAARS as well as either a WURS total score of ≥ 30 or more than five symptoms in either of the two DSM-IV domains (i.e., inattention and hyperactivity/impulsivity) were required for a diagnosis of ADHD (Gerasch et al., 2016). One patient fulfilled this criterion. Hence, in total, four patients with GTS were diagnosed with comorbid ADHD or OCD.

Participants also completed a short version of the Brief Symptom Inventory, the BSI-18 (Derogatis, 2001) as a measure of general psychological distress in three domains (depression, anxiety, somatization), the Beck’s Depression Inventory II (BDI-II; Beck et al., 1996) for the assessment of depressive symptoms, and a short version of the Barratt Impulsiveness Scale, the BIS-Brief (Steinberg et al., 2013). We further used a German vocabulary test (Wortschatztest, WST; Schmidt and Metzler, 1992) to obtain an estimate of premorbid crystallized intelligence. As a global measure of cognitive functioning, we administered the Montreal Cognitive Assessment (MoCA; Nasreddine et al., 2005). The number of completed categories and the number of perseverative errors on a manual version of the WCST, the M-WCST (Schretlen, 2010), were analyzed as well to facilitate the comparison with the studies included in the meta-analysis presented above.

#### 3.1.3 Task and procedure

In accordance with previous studies (Lange et al., 2015a, Lange et al., 2015b, Lange et al., 2016e), we used an adaptation of the computerized card-sorting paradigm introduced by Barceló (2003) that we refer to as cWCST. The cWCST was designed using the Presentation^®^ software and displayed on a 24 inch flat screen at a viewing distance of 120 cm. Responses were collected by a Cedrus^®^ response pad (RB830).

**Fig. 3:**
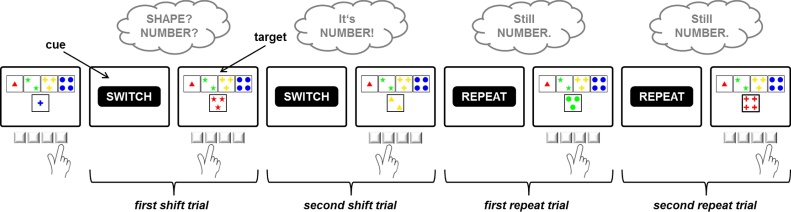
Task dynamics of the computerized Wisconsin Card Sorting Test. Participants were required to match cards according to one of three possible sorting rules (color, shape, number). Task rules switched in an unpredictable manner. Feedback cues following each sorting response indicated whether the applied sorting rule should be maintained or changed on the upcoming trial. Clouds indicate the dynamic updating of task rules as a function of feedback information.

Participants were required to match cards according to one of three possible sorting rules. Target displays consisted of four key cards which appeared invariantly above one stimulus card, all configured around the center of the computer screen (Fig. 3). Stimulus cards varied on three dimensions (color, shape, number), and these dimensions equaled the three viable task rules. None of the 24 different stimulus cards shared more than one attribute with any of the key cards. As a consequence, it was always possible to unambiguously identify the sorting rule applied by the participant (Barceló, 2003, Nelson, 1976).

Participants were instructed that their task would be to match the stimulus card with one of the four key cards in accordance with the appropriate rule. They sorted the cards by pressing one of four keys on the response pad that were mapped to the spatial position of the key cards on the screen. Target displays remained on screen until a key was pressed.

After an interval of 800 ms following participants’ response, a feedback cue was presented for 400 ms, indicating whether the applied sorting rule should be maintained or changed. The German words for “REPEAT” (“BLEIBEN”) and “SHIFT” (“WECHSELN”), displayed in 28 point Arial, were used as feedback cues. Subsequent target stimuli appeared 1200 ms after feedback-cue onset.

Rules changed in an unpredictable manner (Altmann, 2004) after runs of two or more rule repetitions (average run length: 3.5 trials). Participants completed 40 runs involving 39 rule shifts. The average number of trials required to complete these 40 task runs depended on participants’ performance and it did not differ significantly between patients with GTS (*M* = 211.96, *SD* = 19.01) and controls (*M* = 218.54, *SD* = 16.11; *t*[47] = −1.31, *p* = 0.196). Prior to the test sequence, five practice runs were administered. Participants were explicitly informed about the three possible sorting rules, and they were told that the valid rule would change from time to time.

#### 3.1.4 Electrophysiological recordings

Continuous electroencephalogram (EEG) was recorded from 30 active Ag-AgCl electrodes mounted on an actiCap (EASYCAP, Herrsching, Germany) according to the international 10–20 system. BrainVision Recorder version 1.20 software was used (Brain Products, Gilching, Germany). Electrode impedance was kept below 10 kΩ. Electrodes were referenced to FCz electrode. Vertical and horizontal electrooculogram were recorded with two additional electrodes positioned at the suborbital ridge and the external ocular canthus of the right eye, respectively. All channels were amplified (low-pass filter: 70 Hz, time constant: 0.3 s) and digitized at 250 Hz using a BrainAmp amplifier (Brain Products, Gilching, Germany).

#### 3.1.5 Data analysis

For the analyses of performance data and ERP amplitudes, we distinguished between four different trial types (Fig. 3). After a change of the valid cWCST rule, participants encountered a shift feedback cue when they continued to apply the previously correct rule. This first shift cue after a change in task rules initiated a first shift trial. When participants did not shift to the correct rule on the first shift trial, they encountered a second shift feedback cue. The trial initiated by this second shift feedback cue is referred to as second shift trial. As soon as participants had identified the correct new rule after a change in task rules, the first sort according to this rule resulted in a repeat feedback cue. This first repeat feedback cue initiated a first repeat trial. When, in accordance with the cue instruction, participants maintained the sorting rule on the first repeat trial, they encountered a second repeat feedback cue. This second repeat feedback cue initiated a second repeat trial.

Only trials with correct responses were included for RT analysis. RTs shorter than 100 ms or longer than three standard deviations above the mean for each participant were excluded. Mean RTs were subjected to a 2 × 4 mixed ANOVA involving the factors group (GTS vs. controls) and trial type (first shift vs. second shift vs. first repeat vs. second repeat).

For the analysis of response accuracy, we computed the percentages of erroneous responses for each of the four trial types. Error rates were subjected to a 2 × 4 mixed ANOVA involving the factors group (GTS vs. controls) and trial type (first shift vs. second shift vs. first repeat vs. second repeat). Note that errors on the cWCST can occur for a variety of reasons. For example, on the first shift trial, participants might make an erroneous response because they fail to shift away from the previously applied rule or because they switch to the wrong rule. Whereas the first type or error (a perseverative error) is typically regarded as an indicator of set-shifting deficits, the second type of error is rather a sign of an efficient trial-and-error process required by the demands of the cWCST (Barceló, 1999, Nyhus and Barceló, 2009). Similarly, on repeat trials, errors might result from a failure to maintain the previously applied task rule or from a change of the valid task rule that cannot be anticipated by the participant. We have thus added a focused analysis of particular, more narrowly defined types of errors, which have previously been shown to relate to distinct cognitive components of cWCST performance (Lange et al., 2016a, Lange et al., 2016b). Specifically, we distinguished perseverative errors (i.e., sorts according to a rule after shift feedback has indicated that this rule is no longer valid), integration errors (i.e., failures to integrate the relevant information after a rule shift to infer the correct new rule), and set-loss errors (i.e., failures to maintain the correct rule on repeat trials), as indicators of set-shifting, rule-inference, and set-maintenance processes, respectively. The numbers of these error types were subjected to a 2 × 3 mixed ANOVA involving the factors group (GTS vs. controls) and error type (perseverative vs. integration vs. set-loss).

EEG data were evaluated using BrainVision Analyzer 2.0 (Brain Products, Gilching, Germany). After offline filtering (low-pass: 30 Hz, 24 dB/oct; notch: 50 Hz), data were screened for nonstereotyped artifacts (voltage step > 75 μV/ms; activity < 0.5 μV/100 ms). Ocular and muscle artifacts were removed using independent component analysis (Jung et al., 2000). EEGs were re-referenced to a common average reference and segmented into epochs from −200 to 1000 ms relative to the onset of target stimuli and feedback cues. Segments were baseline corrected (baseline: −200 to 0 ms) and averaged after residual artifacts (value difference > 150 μV/200 ms; amplitude < −100 μV or >100 μV) had been rejected. After artifact rejection, the average number of available trials ranged from 21 (for second shift trials) to 38 (for first repeat trials). We provide a complete overview of available trial numbers and grand average ERPs for all channels, conditions, and groups in the supplementary materials.

In line with previous studies investigating the P3 deflections in shifting paradigms, we analyzed the P3 at a frontal (Fz) and a parietal (Pz) electrode to dissociate the anterior and posterior portions of this component (Barceló et al., 2002). At each of these electrodes, we determined individual P3 peak latencies by searching for the local maximum in positive-going ERP activity in the time window from 250 to 500 ms after the onset of cue and target stimuli, separately for the four trial types. Mean P3 amplitudes were measured in a 120 ms (±60 ms) interval around individual peak latencies. Mean P3 amplitudes were subjected to a 2 × 2 × 2 × 4 mixed ANOVA involving the factors group (GTS vs. controls), locking event (cues vs. target), recording site (Fz vs. Pz), and trial type (first shift vs. second shift vs. first repeat vs. second repeat). The analysis of interactions involving the factor group in this admittedly complex design allows examining whether any observed GTS-related ERP alterations are specific to a particular locking event, recording site, and/or trial type.

Significant main effects or interactions involving the factor group were followed up by rerunning the respective analysis with the inclusion of clinical scales assessing comorbid symptoms as covariates. Specifically, we added the BDI-II sum score, the three BSI-18 scales, the DSM-IV symptom list for ADHD, the eight CAARS scales, and the WURS-k as covariates to assess whether the GTS diagnosis significantly contributes to the respective group differences over and above the presence of affective or ADHD symptoms. All analyses were carried out using SPSS 23.0 (IBM, Armonk, NY). The level of significance was set to 0.05. Effect sizes for ANOVAs were calculated as partial eta squared (*η_p_*^2^).

### 3.2 Results

#### 3.2.1 Background assessment

The included neuropsychological measures of cognitive functioning did not reveal substantial GTS-related deficits. Patients with GTS and controls did not differ significantly with regard to their scores on the WST (assessing premorbid crystallized intelligence), MoCA (assessing global cognitive functioning), or M-WCST (see Table 3). In contrast, patients with GTS scored significantly higher than healthy control participants on most of the scales assessing symptoms of comorbid psychiatric disorders (see Table 3).

#### 3.2.2 Behavioral data

A 4 (trial type) × 2 (group) ANOVA on participants’ error rates did not reveal any evidence for response accuracy differences between patients with GTS and controls. Neither the main effect of group, *F*[1, 47] = 0.43, *p* = 0.518, *η_p_*^2^ = 0.01, nor the trial type × group interaction, *F*[3, 141] = 2.11, *p* = 0.128, *η_p_*^2^ = 0.04, was statistically significant. There was a significant main effect of trial type, *F*[3, 141] = 228.55, *p <* 0.001, *η_p_*^2^ = 0.83, with low accuracy on first shift trials, high accuracy on first repeat trials, and intermediate levels of accuracy on second shift trials and second repeat trials (see Table 4). Note that many of these errors are due to the task structure of the cWCST, which requires participants to make errors to realize that the valid rule has changed (see Section 3.1.5).

**Table 4:** Performance data from patients with Gilles de la Tourette syndrome (GTS) and control participants.

	Mean response times (*SD*) in ms			
	First shift	Second shift	First repeat	Second repeat
GTS	3083 (1644)	2624 (1812)	1642 (718)	1386 (453)
controls	2126 (855)	1894 (978)	1285 (342)	1164 (293)
effect size *d*	0.74	0.51	0.64	0.59

	Mean error rates (SD) in %			
	First shift	Second shift	First repeat	Second repeat
GTS	53.0 (6.4)	28.1 (17.3)	6.6 (6.6)	31.5 (7.3)
controls	59.4 (8.1)	30.3 (16.8)	5.1 (4.9)	30.3 (8.2)
effect size *d*	−0.87	−0.13	0.26	0.29

	Mean number (SD) of narrowly defined error types		
	perseverative	integration	set-loss
GTS	10.0 (7.7)	10.9 (8.1)	5.0 (4.8)
controls	11.9 (6.8)	13.2 (8.0)	5.1 (4.2)
effect size *d*	−0.27	−0.29	−0.01

The focused analysis of particular error types yielded similar results. Participants committed more perseverative and integration errors than set-loss errors, as indicated by a significant main effect of error type, *F*[2, 94] = 42.21, *p <* 0.001, *η_p_*^2^ = 0.47, in a 3 (error type) × 2 (group) ANOVA. However, the main effect of group, *F*[1, 47] = 0.73, *p* = 0.397, *η_p_*^2^ = 0.02, and the error type × group interaction, *F*[2, 94] = 1.14, *p* = 0.322, *η_p_*^2^ = 0.02, were not significant.

In contrast, the groups differed significantly with regard to response latency, as indicated by a significant main effect of group, *F*[1, 47] = 6.13, *p* = 0.017, *η_p_*^2^ = 0.12 in a 4 (trial type) × 2 (group) ANOVA on participants’ RTs. On average, patients with GTS responded 556 ms more slowly than controls. This main effect of group did not remain significant when we included the sum scores on the BDI-II (*p* = 0.092), the three BSI-18 scales (*p* = 0.187), the DSM-IV symptom list for ADHD (*p* = 0.272), the eight CAARS scales (*p* = 0.063), and the WURS-k (*p* = 0.056) as covariates. The main effect of trial type was significant as well, *F*[3, 141] = 37.01, *p <* 0.001, *η_p_*^2^ = 0.44. Response latencies gradually decreased from first shift to second repetition trials (see Table 4). The trial type × group interaction was not significant, *F*[3, 141] = 2.63, *p* = 0.098, *η_p_*^2^ = 0.05.

#### 3.2.3 ERP data

**Fig. 4:**
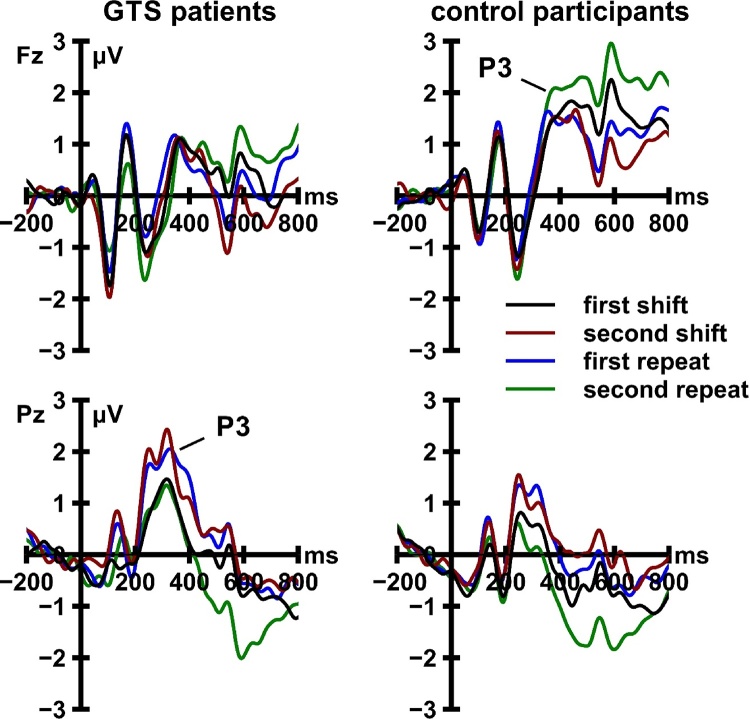
Cue-locked event-related potential activity recorded from patients with Gilles de la Tourette syndrome (GTS) and control participants. ERP data are low-pass filtered (12 Hz, 24 dB/oct) for display purposes only.

**Fig. 5:**
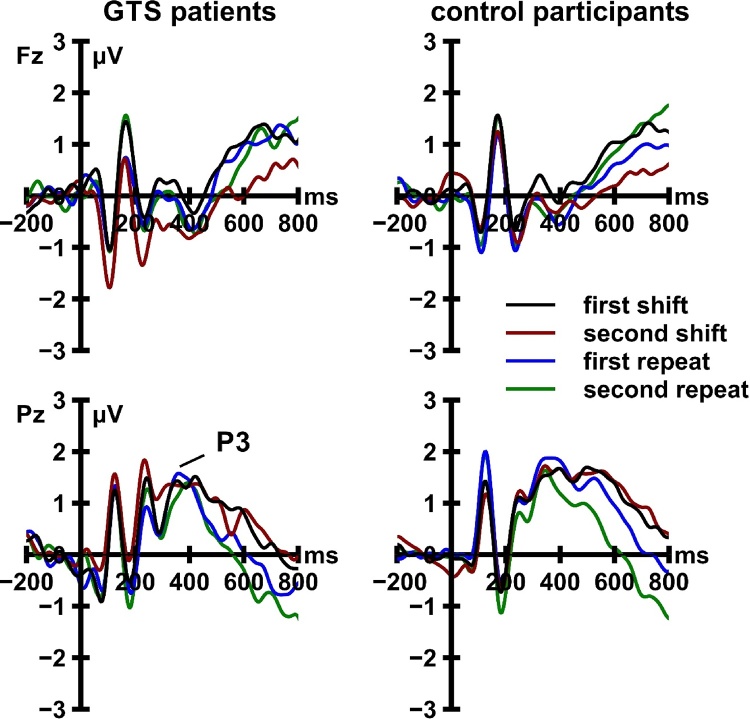
Target-locked event-related potential activity recorded from patients with Gilles de la Tourette syndrome (GTS) and control participants. ERP data are low-pass filtered (12 Hz, 24 dB/oct) for display purposes only.

**Fig. 6:**
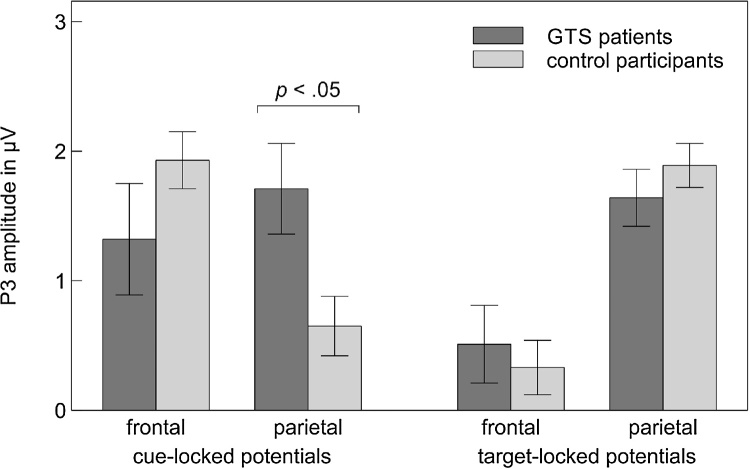
Cue-locked and target-locked P3 amplitudes, pooled across trial types, as a function of recording site and group. Error bars indicate standard error of the mean. GTS = Gilles de la Tourette syndrome.

Cue-locked and target-locked ERP activity is displayed in Fig. 4, Fig. 5, respectively. The 2 (locking event) × 2 (recording site) × 4 (trial type) × 2 (group) mixed ANOVA revealed significant main effects of locking event, *F*[1, 47] = 6.94, *p* = 0.011, *η_p_*^2^ = 0.13, and trial type, *F*[3, 141] = 3.38, *p* = 0.028, *η_p_*^2^ = 0.07. Both main effects were moderated by recording site (locking event × recording site: *F*[1, 47] = 22.32, *p <* 0.001, *η_p_*^2^ = 0.32; trial type × recording site: *F*[3, 141] = 5.37, *p* = 0.002, *η_p_*^2^ = 0.10). Most crucially, the ANOVA revealed a significant three-way interaction between locking event, recording site, and group, *F*[1, 47] = 7.60, *p* = 0.008, *η_p_*^2^ = 0.14 (Fig. 6). This interaction remained significant when we included the sum scores on the BDI-II (*p* = 0.026), the three BSI-18 scales (*p* = 0.027), the DSM-IV symptom list for ADHD (*p* = 0.040), the eight CAARS scales (*p* = 0.012), and the WURS-k (*p* = 0.021) as covariates. Follow-up analyses revealed that cue-locked, *F*[1, 47] = 5.22, *p* = 0.027, *η_p_*^2^ = 0.10, but not target-locked, *F*[1, 47] = 0.84, *p* = 0.364, *η_p_*^2^ = 0.02, P3 amplitudes were significantly modulated by the interaction of recording site and group. At electrode Fz, cue-locked P3 amplitudes did not differ significantly between patients with GTS and HC, *t*[47] = −1.25, *p* = 0.221. At electrode Pz, cue-locked P3 amplitudes were significantly larger in patients with GTS than in HC, *t*[47] = 2.56, *p* = 0.014.

#### 3.2.4 Post-hoc correlation analysis

We provide a table depicting the bivariate correlations between the rating scales administered during background assessment and the performance and ERP measures obtained from the cWCST in the supplementary materials. Although the exploratory nature of these analyses and the large number of examined associations do not permit drawing strong inferences, some aspects of the data might be of interest for future follow-up investigations. First, RTs in patients with GTS were positively related to inattentive symptoms as measured with the DSM-IV symptom list for ADHD. Second, the number of perseveration, integration, and set-loss errors committed by patients with GTS on the cWCST increased with increasing BIS scores. Third, cue-locked P3 amplitudes at electrode Pz were particularly large in patients with GTS and increased YBOCS scores.

## 4 General discussion

Our meta-analysis revealed the first conclusive evidence for significant WCST deficits in patients with GTS. However, this effect was mainly driven by the studies examining WCST performance in underage individuals. While performance deficits were of substantial magnitude in children and adolescents with GTS, they appeared to dissolve in samples of adult patients. Our analysis of event-related neural activity in adult patients with GTS provided first insights into potential neural mechanisms underlying this age-dependent normalization of WCST performance. We observed a marked increase of cue-locked parietal P3 amplitudes in adult patients with GTS as compared to matched controls, which is suggestive of the recruitment of additional proactive control resources. The GTS-related enhancement of cue-locked P3 activity might thus be a correlate of those neural adaptations that allow adult patients with GTS to successfully complete complex neuropsychological tests of cognitive flexibility.

At first sight, our meta-analytical results contrast with the conclusion of Eddy et al. (2009) who did not find evidence in support of GTS-related WCST deficits in a review of the neuropsychological literature. However, rather than being a sign of true inconsistency, our observation of significant WCST deficits in patients with GTS merely illustrates the superior sensitivity of meta-analytical methods in contrast to narrative reviews (Demakis, 2006). As displayed in Fig. 2, many of the confidence intervals surrounding the effect sizes that we extracted from the original studies included the value of zero. When considered in isolation, the non-significant results from such an original study do not allow inferring anything but the absence of evidence for GTS-related deficits in the WCST. This seemingly inconclusive information might, however, be part of a larger empirical pattern that supports the presence of non-negligible WCST deficits in patients with GTS. Our meta-analysis identified this pattern and, at the same time, served to detect an important moderator of WCST performance in patients with GTS. While it is important to note that this conclusion is based on cross-sectional data, it appears that WCST performance in patients with GTS is subject to substantial age-related changes: GTS-related WCST deficits are large in children and adolescents, significantly decrease as patients mature, and are indistinguishable from zero in adult patients. At this point, we do not wish to overstate the magnitude or generality of WCST deficits in young persons with GTS. The large mean effect size obtained for the category measure in this age group was based on seven studies, five of which did not include a control group and compared patient data to a normative sample instead (which might have led to biased effect-size estimates). The two studies that did include a control group reported large effect sizes (0.83 and 1.14), but they differed from the majority of other neuropsychological studies on GTS in exclusively including participants from East Asian countries. More neuropsychological studies providing unbiased reports of WCST performance data in patients with GTS are required to examine the potential moderating role of such aspects of the study design and population.

In line with our meta-analytical results, the adult patients with GTS participating in our ERP study did not differ significantly from a matched control group with regard to M-WCST performance. Similarly, patients with GTS did not commit an increased number of errors on the computerized version of the WCST. In contrast, patients’ RTs were significantly prolonged, which might suggest that patients with GTS require more time to adequately respond to the rule-shifting demands of the cWCST. However, GTS-related RT prolongation was found on all trial types, indicating that patients did not have specific difficulties with shifting cognitive sets. Instead, the speed of motor output in general seems to be slowed in patients with GTS. Similar data were observed by Shephard et al. (2016a), who concluded that the GTS-related slowing of motor responses might reflect a compensatory mechanism facilitating the control of tics. Note, however, that the group difference in RTs observed in our study did not remain significant when we included psychometric questionnaires assessing comorbid symptoms as covariates in our analysis. In addition, our post-hoc correlation analyses revealed an association between responses times and a facet of ADHD symptoms in patients in GTS. Hence, at this point, we are not able to conclude that response slowing in patients with GTS is specific to the presence of tic symptoms.

The most important result of our ERP study is the increase in cue-locked parietal P3 activity in patients with GTS. Cue-related P3 deflections are commonly considered as a neural correlate of proactive cognitive control processes (Barceló et al., 2002, Kopp et al., 2014). Recall that the feedback cues on the cWCST did not require any overt motor response; they merely informed participants about whether or not to switch the applied sorting rule on the upcoming trial. The neural responses elicited by those cues are thus unlikely to be related to reactive cognitive processing at the response-selection stage. They rather reflect preparatory or anticipatory cognitive processes such as the proactive activation of task rules (Barceló et al., 2002). Our results suggest that the recruitment of proactive cognitive control resources is facilitated rather than impaired in patients with GTS.

This interpretation is consistent with the findings reported by Jackson and colleagues (Jackson et al., 2007, Jung et al., 2015, Mueller et al., 2006), who inferred a GTS-related enhancement of cognitive control from patients’ behavior on an oculomotor switching task (see also Jackson et al., 2011, for related brain imaging data). Jackson et al. (2011) proposed that GTS is associated with “compensatory changes in brain structure and function” (p. 584), which can already be observed in young patients. These neural alterations might allow patients to control their motor symptoms, but also to exert increased levels of cognitive control in shifting paradigms. Our findings add to this knowledge by illustrating that the neural correlates of enhanced cognitive control in adults with GTS can be measured at the scalp surface. In addition, our ERP analysis allowed us to decompose different modes of cognitive control. By demonstrating that GTS-related cognitive changes affect proactive rather than reactive cognitive-control processes, our data complement the insights from behavioral studies and contribute to a more fine-grained understanding of executive functioning in GTS.

The study of executive functions in general or cognitive flexibility in particular in patients with GTS might have important implications for the design and administration of behavioral treatments for tic disorders. For example, habit reversal training techniques involve learning to execute an alternative response when patients detect a premonitory urge to tic (Himle et al., 2006). Intact cognitive flexibility might be a necessary prerequisite for behavior change and the reversal of habits in GTS. However, research on the neuropsychological predictors of patients’ response to habit reversal learning is scarce (Deckersbach et al., 2006) and the role of cognitive flexibility in determining treatment response in GTS remains to be examined.

### 4.1 Limitations

One important limitation of our present analysis is its exclusive reliance on cross-sectional data. While our meta-analysis showed a clear moderating effect of age on GTS-related WCST deficits, interpreting this effect as evidence for an age-related normalization of cognitive flexibility in GTS does involve some degree of speculation. Due to the lack of longitudinal studies that monitor patients’ WCST performance as they mature, we cannot conclusively demonstrate that the WCST deficits shown by a particular patient disappear when this patient reaches adulthood. Similarly, our approach of studying the ERP correlates of WCST performance in adult patients with GTS to shed light on the neural mechanisms that underlie the proposed age-related normalization is clearly limited. It would certainly be desirable to complement our results with comparable data obtained from children with GTS in the future. Ideally, such a future investigation could follow the example of a study recently published by Eichele et al. (2017) who examined the development of behavioral and ERP indicators of conflict resolution and performance monitoring in children with GTS over a period of 4.5 years.

Another aspect of our results that requires further investigation using optimized research designs is the potential relationship between clinical variables and behavioral and electrophysiological cWCST measures. Our post-hoc correlation analysis revealed a potentially interesting correlation between cue-locked parietal P3 amplitudes and OCD symptoms in patients with GTS. However, as our study was not designed to characterize the relationship between ERPs and clinical variables, the size of our sample does not allow for a conclusive test of this correlation. Specifically, it is not possible to clarify whether the observed P3 alteration is more closely related to OCD symptoms than to tic severity. Large-sample replication studies are required to answer this particular question and, more generally, to determine the role of comorbid OCD symptoms for WCST performance deficits in GTS. These studies might also benefit from focusing on the role of impulsiveness, a trait that was related to patients’ performance on the cWCST in the present study (see supplementary materials) and in a previous study involving patients with dystonia (Lange et al., 2016d). By contributing to an improved understanding of impulsiveness and impulse control in GTS, such research would also address a GTS-related problem of high clinical relevance (Frank et al., 2011, Mathews et al., 2004, Sambrani et al., 2016).

Finally, it is important to acknowledge that cognitive flexibility is likely to be a multifaceted construct, and different aspects of cognitive flexibility might differ in their relevance for patients with GTS. In addition to the WCST results we analyzed here, GTS has been reported to be associated with significantly improved (Mueller et al., 2006) and significantly impaired (Watkins et al., 2005) performance on other tests of cognitive flexibility. Future work is required to compare and contrast facets of cognitive flexibility and their clinical importance in patients with GTS. Similarly, it would be desirable to combine multiple physiological indicators of executive functions to obtain a more comprehensive understanding of the neural mechanisms underlying cognitive flexibility in GTS. In this context, comparative analyses of multiple ERP correlates of executive functioning (e.g., Seer et al., 2016) or simultaneous EEG and fMRI recordings (Ullsperger and Debener, 2010) might be particularly promising.

## 5 Conclusion

GTS appears to involve deficits in cognitive flexibility, which are significantly modulated by age-related changes. Adults with GTS may be able to successfully complete complex tasks of cognitive flexibility by enhanced recruitment of proactive cognitive control mechanisms. The study of the electrophysiological correlates of these cognitive alterations opens a new window onto patterns of neural reorganization in GTS.

## Conflict of Interest

None.
