# Supplementary material for: Cognitive flexibility and its electrophysiological correlates in Gilles de la Tourette syndrome
Source: Dev Cogn Neurosci. 2017 Aug 18;27:78–90. doi: 10.1016/j.dcn.2017.08.008 (PMC6987949; doi:10.1016/j.dcn.2017.08.008)
Supplement: Supplementary file 1 [file mmc1.pdf]

cue-locked event-related potentials

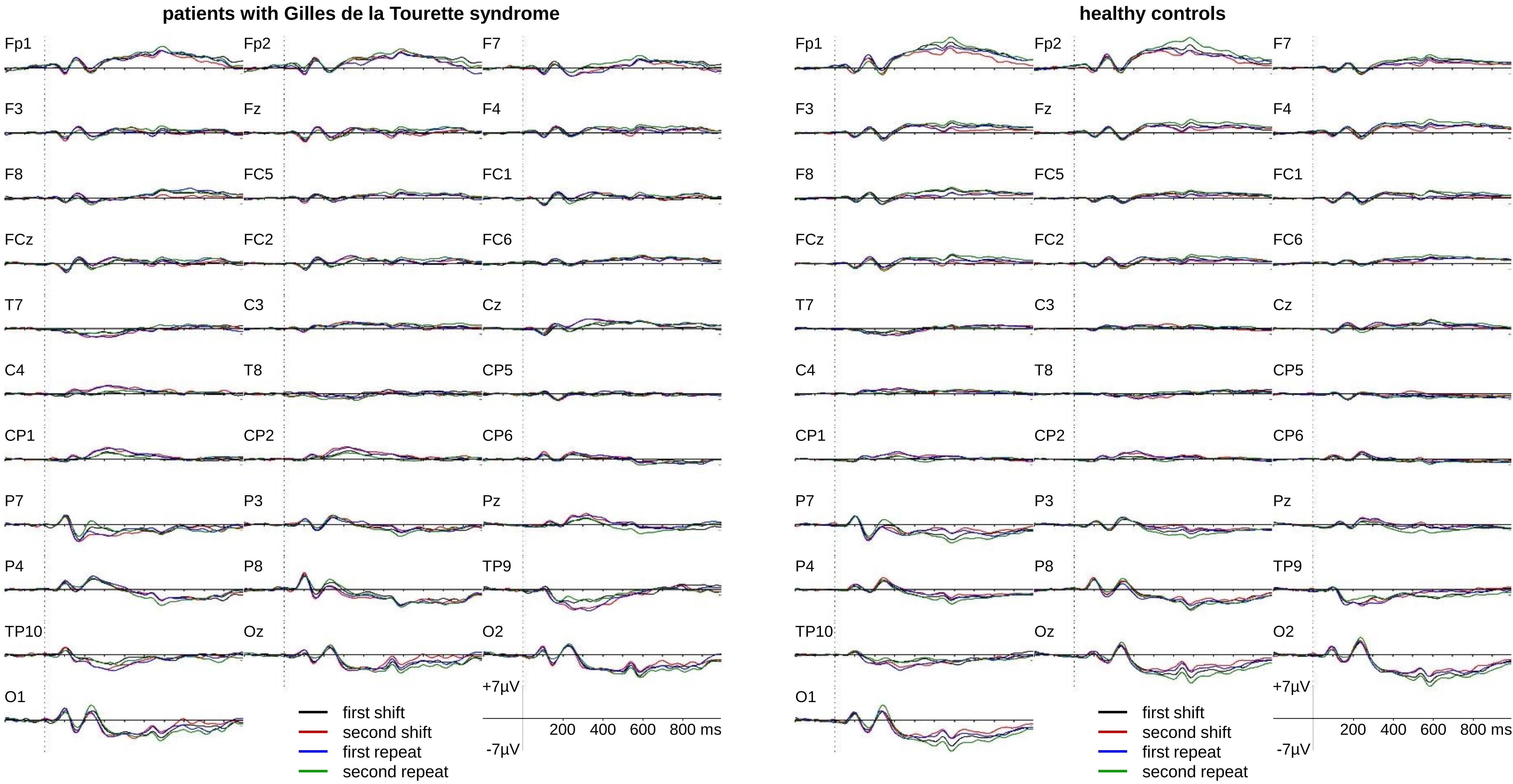

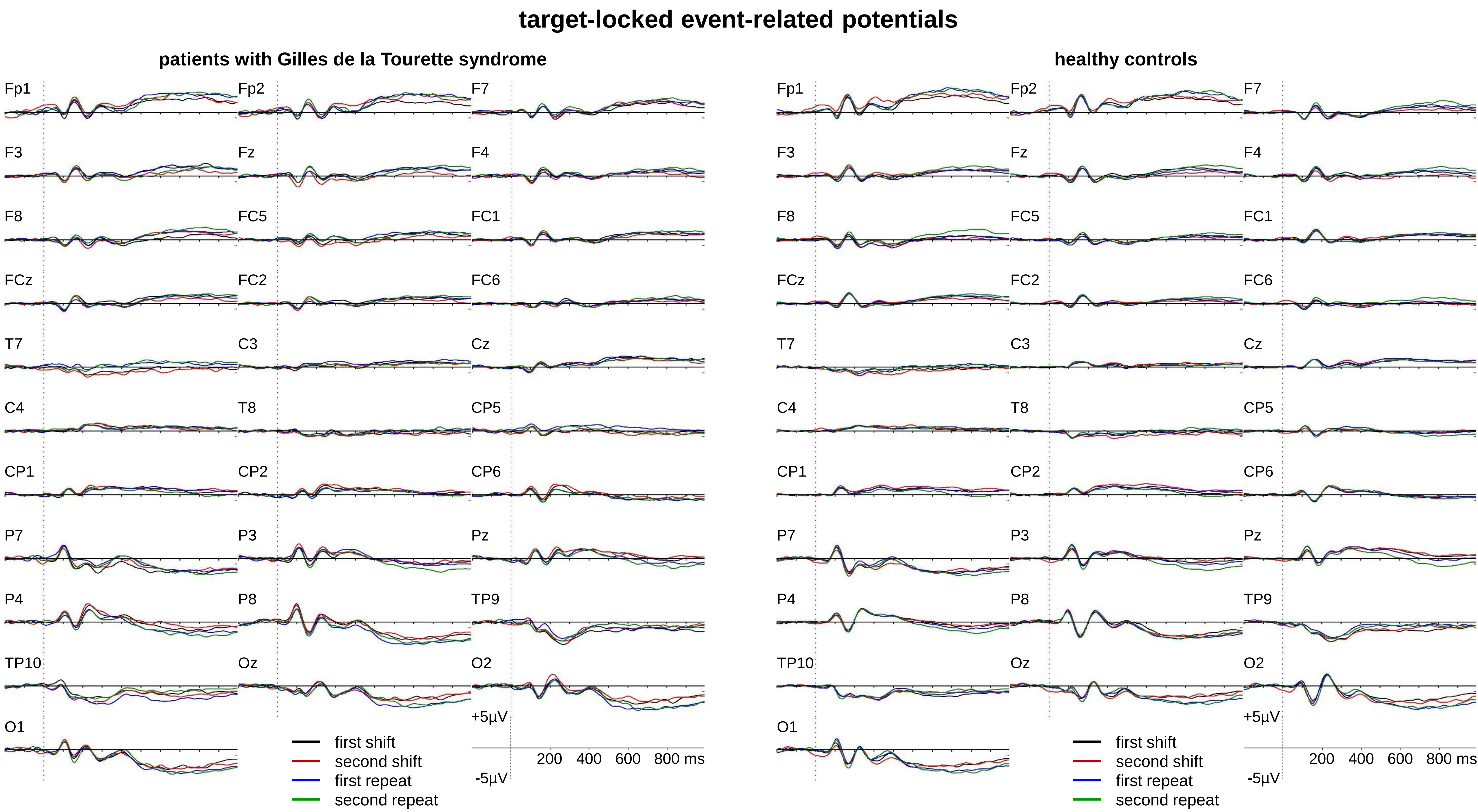
target-locked event-related potentials
patients with Gilles de la Tourette syndrome
healthy controls
Fp1
Fp2
F7
F3
Fz
F4
F8
FC5
FC1
FCz
FC2
FC6
T7
C3
Cz
C4
T8
CP5
CP1
CP2
CP6
P7
P3
Pz
P4
P8
TP9
TP10
Oz
O2
O1
+5µV
-5µV
200
400
600
800 ms
first shift
second shift
first repeat
second repeat
