# Supplementary material for: Cognitive flexibility and its electrophysiological correlates in Gilles de la Tourette syndrome
Source: Dev Cogn Neurosci. 2017 Aug 18;27:78–90. doi: 10.1016/j.dcn.2017.08.008 (PMC6987949; doi:10.1016/j.dcn.2017.08.008)
Supplement: Supplementary file 2 [file mmc2.pdf]

Supplementary table 1.
Pearson correlation coefficients for the associations between clinical rating scales and the performance and event-related potential measures obtained from the computerized version of Wisconsin Card Sorting Test in patients with Gilles de la Tourette syndrome

	cue-locked P3 Fz	cue-locked P3 Pz	target-locked P3 Fz	target-locked P3 Pz	Mean response time	Mean error rate	perseve-rative errors	inte-gration errors	set-loss errors
YGTSS	-.10	.33	-.23	-.10	.24	-.24	-.26	-.23	-.07
ATQ	-.06	.22	-.16	.00	.42	-.05	-.07	-.02	.05
PUTS	-.10	.33	-.11	.28	-.12	-.13	-.15	-.19	-.20
YBOCS	-.17	.51*	.12	.03	.19	-.16	-.15	-.03	-.04
BIS-Brief	.14	.00	.07	.22	.31	.40	.46*	.46*	.69*
BDI-II	-.01	.34	.11	.18	.23	.27	.24	.32	.25
BSI-18 Global severity	-.02	.23	-.04	.03	.36	.40	.34	.38	.38
BSI-18 Anxiety	-.02	.25	-.04	-.15	.33	.25	.18	.24	.20
BSI-18 Depression	.03	.00	-.14	.18	.24	.47*	.42*	.36	.37
BSI-18 Somatization	-.07	.33	.09	.07	.33	.31	.30	.39	.42*
CAARS inattention	-.06	.23	.08	.62*	.14	-.01	.20	.12	.18
CAARS hyperactivity	-.03	.24	.28	.33	.24	.10	.35	.33	.30
CAARS impulsivity	-.04	-.01	-.24	.16	.11	.34	.49*	.32	.37
CAARS self-concept	-.03	-.02	-.17	.23	.01	.32	.36	.37	.15
CAARS inattentive symptoms	-.24	.12	-.07	.22	.31	.28	.41	.40	.42*
CAARS hyperactive-impulsive symptoms	.03	.18	.23	.39	.14	.12	.37	.26	.28
CAARS ADHD symptoms	-.11	.17	.08	.36	.26	.22	.43*	.37	.40
CAARS ADHD index	-.06	.10	-.10	.23	.17	.32	.48*	.39	.42*
WURS-k	-.15	.30	-.01	-.09	.32	.27	.31	.37	.37
DSM-IV list attention	-.08	.06	-.08	-.06	.57*	.28	.32	.38	.41
DSM-IV list hyperactivity	.04	-.03	.00	-.04	.36	.27	.31	.27	.43*

*p < .05

Supplementary table 2.
Pearson correlation coefficients for the associations between clinical rating scales and the performance and event-related potential measures obtained from the computerized version of Wisconsin Card Sorting Test in healthy control participants

	cue-locked P3 Fz	cue-locked P3 Pz	target-locked P3 Fz	target-locked P3 Pz	Mean response time	Mean error rate	perseve-rative errors	inte-gration errors	set-loss errors
YGTSS	n/a	n/a	n/a	n/a	n/a	n/a	n/a	n/a	n/a
ATQ	n/a	n/a	n/a	n/a	n/a	n/a	n/a	n/a	n/a
PUTS	n/a	n/a	n/a	n/a	n/a	n/a	n/a	n/a	n/a
YBOCS	n/a	n/a	n/a	n/a	n/a	n/a	n/a	n/a	n/a
BIS-Brief	-.06	.12	.01	-.25	.41*	.02	-.12	.14	.22
BDI-II	.22	-.06	-.04	-.11	.25	-.03	.16	.03	.08
BSI-18 Global severity	-.01	.09	.13	-.18	.41*	.10	.09	.19	.11
BSI-18 Anxiety	-.01	.21	.06	-.14	.37	.27	.19	.33	.17
BSI-18 Depression	-.13	-.06	-.08	-.17	.27	-.13	-.08	.10	-.04
BSI-18 Somatization	.09	.02	.30	-.13	.33	.03	.05	-.01	.10
CAARS inattention	.12	-.28	.08	-.42*	.23	-.04	-.06	.01	.17
CAARS hyperactivity	-.03	.23	.05	-.27	.53*	.01	-.18	-.03	.01
CAARS impulsivity	.17	-.13	.00	-.37	.39	-.07	-.13	-.09	.02
CAARS self-concept	.14	-.09	.02	-.36	.41*	-.11	-.21	-.10	.13
CAARS inattentive symptoms	.22	-.06	.06	-.37	.57*	.01	-.05	.13	.26
CAARS hyperactive-impulsive symptoms	.03	-.02	-.06	-.38	.39	.16	-.07	.11	.26
CAARS ADHD symptoms	.13	-.04	.04	-.45*	.53*	.13	-.03	.16	.30
CAARS ADHD index	.22	-.14	.03	-.41*	.42*	-.07	-.22	-.07	.16
WURS-k	-.01	.19	.18	.08	.26	-.19	-.20	-.22	-.24
DSM-IV list attention	.02	.25	.15	-.19	.40	.21	-.05	.17	.09
DSM-IV list hyperactivity	.11	.27	.16	.08	.17	.06	.17	.09	.24

*p < .05

Supplementary table 3.

Trial numbers available for the analysis of event-related potentials after artifact rejection from patients with Gilles de la Tourette syndrome (GTS) and control participants

	mean number of included trials (*SD*)							
	cue-locked potentials				target-locked potentials			
	First shift	Second shift	First repeat	Second repeat	First shift	Second shift	First repeat	Second repeat
GTS	35.87 (4.12)	19.30 (3.47)	36.70 (3.36)	34.78 (3.73)	35.70 (4.26)	19.61 (3.04)	36.17 (3.47)	34.70 (4.39)
controls	37.77 (1.70)	22.81 (3.05)	38.46 (1.58)	36.23 (3.15)	37.65 (2.04)	22.69 (3.23)	37.85 (2.41)	36.12 (3.25)
